# Alcohol Consumption, Types of Alcohol, and Parkinson’s Disease

**DOI:** 10.1371/journal.pone.0066452

**Published:** 2013-06-19

**Authors:** Rui Liu, Xuguang Guo, Yikyung Park, Jian Wang, Xuemei Huang, Albert Hollenbeck, Aaron Blair, Honglei Chen

**Affiliations:** 1 Epidemiology Branch, National Institute of Environmental Health Sciences, Research Triangle Park, North Carolina, United States of America; 2 Westat Inc., Research Triangle Park, North Carolina, United States of America; 3 Division of Cancer Epidemiology and Genetics, National Cancer Institute, Rockville, Maryland, United States of America; 4 Department of Neurology, Huashan Hospital, Fudan University, Shanghai, P. R. China; 5 Departments of Neurology, Pennsylvania State University-Milton S. Hershey Medical Center, Hershey, Pennsylvania, United States of America; 6 AARP, Washington, District of Columbia, United States of America; Brigham & Women's Hospital, and Harvard Medical School, United States of America

## Abstract

**Background:**

The epidemiologic evidence on alcohol consumption and Parkinson’s disease (PD) is equivocal. We prospectively examined total alcohol consumption and consumption of specific types of alcoholic beverage in relation to future risk of PD.

**Methods:**

The study comprised 306,895 participants (180,235 male and 126,660 female) ages 50–71 years in 1995–1996 from the NIH-AARP Diet and Health Study. Consumption of alcoholic beverages in the past 12 months was assessed in 1995–1996. Multivariate odds ratios (OR) and 95% confidence intervals (CI) were obtained from logistic regression models.

**Results:**

A total of 1,113 PD cases diagnosed between 2000 and 2006 were included in the analysis. Total alcohol consumption was not associated with PD. However, the association differed by types of alcoholic beverages. Compared with non-beer drinkers, the multivariate ORs for beer drinkers were 0.79 (95% CI: 0.68, 0.92) for <1 drink/day, 0.73 (95% CI: 0.50, 1.07) for 1–1.99 drinks/day, and 0.86 (95% CI: 0.60, 1.21) for ≥2 drinks/day. For liquor consumption, a monotonic increase in PD risk was suggested: ORs (95% CI) were 1.06 (0.91, 1.23), 1.22 (0.94, 1.58), and 1.35 (1.02, 1.80) for <1, 1–1.99, and ≥2 drinks/day, respectively (P for trend <0.03). Additional analyses among exclusive drinkers of one specific type of alcoholic beverage supported the robustness of these findings. The results for wine consumption were less clear, although a borderline lower PD risk was observed when comparing wine drinkers of 1–1.99 drinks/day with none drinkers (OR = 0.74, 95% CI: 0.53, 1.02).

**Conclusions:**

Our results suggest that beer and liquor consumption may have opposite associations with PD: low to moderate beer consumption with lower PD risk and greater liquor consumption with higher risk. These findings and potential underlying mechanisms warrant further investigations.

## Introduction

Alcohol drinking is a common lifestyle choice that can have significant and complex behavioral, medical and public health consequences [1], [2]. While heavy alcohol use is undoubtedly detrimental to health, light to moderate alcohol consumption has been linked to lower risk of cardiovascular disease [1] and more recently to lower risk of cognitive decline and dementia [3], [4]. Little, however, is known about alcohol drinking and Parkinson’s disease (PD). Like smoking and coffee consumption, excessive alcohol drinking may be a feature of a risk-taking personality that has been hypothesized to be associated with lower PD risk [5]. Moreover, consumption of alcoholic beverages, particularly beer, elevates plasma urate [6]; urate is a potent free-radical scavenger that has been linked to lower PD risk [7]–[10] and slower PD progression [11], [12]. Given the paucity of existing epidemiologic evidence, we examined the consumption of total and specific types of alcoholic beverages and PD risk in a large US cohort of older adults.

## Methods

### Study Population and PD Case Identification

The NIH-AARP Diet and Health Study conducted a comprehensive baseline survey on diet and lifestyle in 1995–1996 and recruited 566,398 AARP members (ages 50 to 71) from six US states (California, Florida, North Carolina, Pennsylvania, New Jersey, and Louisiana) and two metropolitan areas (Atlanta and Detroit) [13]. AARP, formerly known as the American Association of Retired Persons, is a non-profit organization with members of 50 years or older across the US. Between 2004 and 2006, a follow-up survey was mailed to surviving participants to update lifestyle exposures and to ascertain the occurrence of major chronic diseases, including PD. Participants reported lifetime diagnosis of PD and the year of diagnosis in the following categories: before 1985, 1985–1994, 1995–1999, or 2000 to present. A total of 318,257 participants (187,496 men and 130,761 women) participated in the follow-up survey and were therefore included in the current analysis. Of these participants, 2,432 reported a PD diagnosis and 315,825 did not. We were concerned that some PD patients might have altered their dietary behaviors and lifestyle even before diagnosis due to nonmotor or subtle motor symptoms (i.e. reverse causality), and therefore excluded all cases who reported a PD diagnosis before the year 2000 (n = 1,094) from the analyses. We further excluded 214 self-identified PD patients whose diagnosis was later contradicted by themselves or by their treating physicians in the validation study described below. In addition, we also excluded 9 cases with extreme energy intakes (defined as values more than 2 interquartile ranges above the 75^th^ or below the 25^th^ percentile on the log-transformed energy intake distribution), and 2 missing on alcohol consumption. Among participants who did not report a PD diagnosis, we excluded 8,050 participants with missing PD status, 1,682 with extreme energy intake, and 311 with missing information on alcohol consumption. The final analytic dataset included 1,113 self-reported PD cases whose first diagnoses were after 2000, and 305,782 participants without PD.

We validated the accuracy of self-reported PD diagnoses in conjunction with DNA collection for PD genetic research. The details of this validation have been described previously [14]. Briefly, we first asked potential PD patients to confirm their earlier self-reports and then asked their treating physicians to complete a diagnostic questionnaire and to provide a copy of the patient’s medical records. The medical records were subsequently reviewed by a movement disorder specialist (X.H.). The diagnosis was considered valid if: 1) the treating neurologist confirmed the diagnosis; or 2) if the medical record included a final PD diagnosis or evidence of two or more cardinal signs of PD (with one being rest tremor or bradykinesia), a progressive course, responsiveness to dopaminergic treatments, and absence of features that suggested an alternative diagnosis. Of the 1,069 physician responses received, 940 (87.9%) PD diagnoses were confirmed. The confirmation rate was similar across years of diagnosis: 83.3% for cases diagnosed before 1985, 92.8% for cases diagnosed in 1985–1994, 87.9% for cases diagnosed in 1995–1999, and 87.2% for cases diagnosed after 2000.

### Exposure Assessment

As part of the cohort’s baseline survey in 1995–1996, consumption of alcoholic beverages was assessed using a validated self-administered 124-item food frequency questionnaire (FFQ) [15]. The FFQ queried consumption of beer during the summer, beer during the rest of the year, liquor or mixed drinks, or wine or wine coolers during the previous 12 months. For each drink, information was sought on frequency of consumption (ten categories ranging from ‘never’ to ‘≥6 times/day’) and three portion sizes (<1, 1–2, >2 drinks). Drinks of alcohol per day were computed for total alcohol and for each type of alcoholic beverage, with one drink of alcoholic beverage defined as one 12-fluid-ounce beer, one 5-fluid-ounce glass of wine, or one 1.5-ounce shot of liquor, based on the US Department of Agriculture’s Food Guide Pyramid [16]. Each drink contains approximately 13 grams of alcohol. Data on coffee and other caffeine containing drinks or foods were also collected as part of the dietary survey. In addition to diet, the baseline survey also collected data on basic demographics and lifestyle factors such as smoking habit, physical activity, and self-evaluated health status.

### Statistical Analysis

In the primary analyses, we categorized the consumption frequencies into drinks per day: none, <1, 1–1.99, 2–2.99, 3–3.99, 4–4.99 and ≥5 for total alcohol, and none, <1, 1–1.99, and ≥2 for individual alcoholic beverages (beer, wine, liquor). Multivariate odds ratios (OR) and 95% confidence intervals (CI) were derived from logistic regression models, first adjusting for age at baseline (in 5-year groups), and then additionally adjusting for sex, race (whites vs. non-whites), education level (less than 12 years, 12 years or completed high school, post-high school or some college, college and post graduate), marital status (married or living as married, widowed, divorced, separated or never married), smoking status (never smokers, past smokers [years since quitting: ≥35, 30–34, 20–29, 10–19, 1–9], and current smokers [cigarettes per day: 1–10, 11–20, >20]), caffeine intake (quintiles), general health status (excellent/very good, good, fair or poor), and physical activity (never/rarely, 1–3 times/month, 1–2 times/week, 3–4 times/week, and ≥5 times/week). Specific types of alcoholic beverage consumption were first analyzed individually and then mutually adjusted for each other. Whenever a monotonic trend was suggested, we examined the statistical significance for a linear trend by including the mid-point of each exposure category as a continuous variable in the regression model. Finally, we conducted several sensitivity and stratified analyses to examine the nature and robustness of our key findings. First, we examined risk associated with specific types of alcoholic beverage in relation to PD among non-drinkers of other types of alcoholic beverages. Second, we stratified the analyses according to known PD risk factors: age (median in years), gender (men and women), smoking status (never and ever [current and past smokers]), and caffeine intake (median). Statistical testing for multiplicative interactions was examined by adding a product term between alcohol intake and potential modifier into the regression model. All statistical analyses were performed using SAS, version 9.1 (SAS Institute Inc., Cary, NC). Significance tests were two-tailed with α = 0.05.

### Standard Protocol Approvals, Registrations, and Patient Consents

Participants consented to the study by returning survey questionnaires. The study protocol was approved by the Institutional Review Board of the National Institute of Environmental Health Sciences and the Special Studies Institutional Review Board of the National Cancer Institute.

## Results

Population characteristics according to total alcohol consumption are shown in **Table 1**. Compared with nondrinkers, alcohol drinkers were more likely to be men, non-Hispanic Whites, have a college education or above, married or living as married, and past or current smokers. They were also more likely to report higher caffeine intake, slightly higher physical activity, and excellent or very good health status.

**Table 1:** Baseline Population Characteristics According to Total Alcohol Consumption Categories, NIH-AARP Diet and Health Study, 1995–2006.

	Total Alcohol (drinks/day)				
	None	<1	1–1.9	2–2.9	≥3
N	66,962	167,066	38,272	11,835	22,760
Mean age in years (SD)	61.7±5.3	61.2±5.4	61.8±5.3	61.4±5.3	61.5±5.3
Men, %	50.0	54.5	71.5	75.6	85.3
Race, %					
Non-Hispanic White	89.2	92.8	95.5	95.8	95.3
Others	9.4	6.3	3.7	3.5	3.9
Missing	1.5	1.0	0.8	0.7	0.7
Education, %					
<12 years	30.6	20.6	14.8	15.0	18.3
High school	10.3	9.5	8.0	8.3	9.1
Post-high/some college	21.7	23.5	21.2	21.9	23.1
College and above	34.3	44.2	54.3	53.2	47.6
Missing	3.1	2.2	1.6	1.6	1.9
Marital status, %					
Married/living as married	66.2	68.9	76.9	77.1	78.1
Widowed	12.9	10.6	6.9	5.9	5.7
Divorced	13.6	14.1	10.5	11.0	10.2
Separated	1.1	1.0	0.8	0.9	0.8
Never Married	5.4	4.9	4.4	4.7	4.8
Unknown	0.8	0.5	0.5	0.5	0.5
Smokers, %					
Never	48.2	40.3	28.6	24.2	17.6
Past	42.1	49.5	60.4	63.6	63.6
Current	8.3	9.0	9.8	11.1	17.4
Missing	1.3	1.2	1.1	1.1	1.3
Caffeine intake (mg/day), Median (IQR)	135.3 (522.4)	232.2 (528.9)	508.5 (520.9)	514.6 (508.9)	526.2 (511.9)
Physical activity, %					
Never or rarely	20.2	15.0	10.9	11.6	15.5
1–3 times/month	13.0	14.0	11.9	12.4	13.6
1–2 times/week	20.1	22.8	22.3	21.8	21.6
3–4 times/week	25.3	28.5	31.2	29.8	27.0
≥5 times/week	20.2	18.9	23.2	23.9	21.6
Missing	1.2	0.7	0.5	0.5	0.6
Self-reported health status, %					
Excellent/very good	46.7	56.9	65.1	65.0	57.5
Good	36.6	33.6	28.5	28.6	33.4
Fair	15.0	8.3	5.4	5.4	8.0
Poor	1.6	1.2	1.0	1.0	1.0
Beer consumption, %					
None	100	34.9	14.5	11.8	7.8
<1	0.0	65.1	71.2	59.3	46.7
≥1	0.0	0.0	14.2	28.9	45.5
Wine consumption, %					
None	100	17.8	12.6	10.9	23.7
<1	0.0	82.2	57.8	36.5	53.5
≥1	0.0	0.0	29.5	52.6	22.8
Liquor consumption, %					
None	100	34.1	17.7	18.3	14.9
<1	0.0	65.9	53.5	45.0	22.5
≥1	0.0	0.0	28.8	36.7	62.6

IQR = inter-quartile range (25%-75%).

Total alcohol consumption at baseline was not related to future PD risk (**Table 2**). When specific types of alcoholic beverages were examined individually, the effects appeared to differ by beverage type. Compared with non-beer drinkers, the multivariate ORs for beer drinkers were 0.79 (95% CI: 0.68, 0.92) for <1 drink/day, 0.73 (95% CI: 0.50, 1.07) for 1–1.99 drinks/day, and 0.86 (95% CI: 0.60, 1.21) for ≥2 drinks/day. In contrast, a monotonic increase in PD risk was observed with greater consumption of liquor (P for trend_ = _0.03). The multivariate-adjusted ORs were 1.06 (95% CI: 0.91, 1.23) for less than 1 drinks of liquor per day, 1.22 (95% CI: 0.94, 1.58) for 1–1.99 drinks per day, and 1.35 (95% CI: 1.02, 1.80) for 2 or more drinks per day. Results for wine consumption were less clear, although a non-significant lowered PD risk was observed when comparing drinkers of 1–1.99 drinks of wine/day with non-wine drinkers (OR = 0.74, 95% CI: 0.53, 1.02).

**Table 2:** Odds Ratios of Parkinson’s Disease According to Consumption of Total Alcohol and Specific Types of Alcoholic Beverages, NIH-AARP Diet and Health Study, 1995–2006.

	Model 1^a^		Model 2^b^	Model 3^c^
Drinks/day	PD/No PD	OR (95% CI)	OR (95% CI)	OR (95% CI)
Total alcohol				
None	262/66,700	1.00	1.00	
<1	570/166,496	0.90 (0.78, 1.05)	0.91 (0.78, 1.06)	
1–1.99	131/38,141	0.86 (0.70, 1.07)	0.82 (0.66, 1.02)	
2–2.99	54/11,781	1.20 (0.89, 1.61)	1.13 (0.84, 1.53)	
3–3.99	35/7,469	1.22 (0.85, 1.73)	1.15 (0.81, 1.65)	
4–4.99	18/4,145	1.13 (0.70, 1.82)	1.06 (0.65, 1.72)	
≥5	43/11,050	1.01 (0.73, 1.39)	0.92 (0.66, 1.28)	
P for trend		0.20	0.63	
Beer				
None	493/133,575	1.00	1.00	1.00
<1	552/153,056	1.00 (0.88, 1.12)	0.84 (0.74, 0.96)	0.79 (0.68, 0.92)
1–1.99	31/9,153	0.97 (0.68, 1.40)	0.79 (0.54, 1.14)	0.73 (0.50, 1.07)
≥2	37/9,998	1.10 (0.79, 1.54)	0.92 (0.65, 1.29)	0.86 (0.60, 1.21)
P for trend		0.61	0.86	0.78
Wine				
None	397/107,745	1.00	1.00	1.00
<1	644/175,397	1.01 (0.89, 1.15)	1.01 (0.89, 1.15)	1.07 (0.92, 1.25)
1–1.99	43/16,145	0.71 (0.52, 0.97)	0.69 (0.50, 0.95)	0.74 (0.53, 1.02)
≥2	29/6,495	1.26 (0.86, 1.84)	1.24 (0.84, 1.81)	1.31 (0.89, 1.94)
P for trend		0.78	0.66	0.82
Liquor				
None	493/135,762	1.00	1.00	1.00
<1	484/140,525	0.98 (0.87, 1.11)	0.98 (0.86, 1.12)	1.06 (0.91, 1.23)
1–1.99	74/16,683	1.12 (0.88, 1.43)	1.10 (0.86, 1.41)	1.22 (0.94, 1.58)
≥2	62/12,812	1.28 (0.98, 1.67)	1.23 (0.94, 1.62)	1.35 (1.02, 1.80)
P for trend		0.03	0.08	0.03

CI = confidence interval; OR = odds ratio; PD = Parkinson’s disease.

a Adjusted for age.

b Additional adjustment for sex, race, education, marital status, smoking, caffeine intake, physical activity, and self-evaluated health status.

c Based on Model 2, individual types of alcoholic beverages were adjusted simultaneously.

When restricted the analyses to exclusive drinkers of a specific type of alcoholic beverage (**Table 3**), significant monotonic trends were observed for both beer drinkers (P for trend = 0.05) and liquor drinkers (P for trend = 0.02). Compared with non-alcohol drinkers, those who consumed one or more drinks of beer per day had 59% lowered risk of PD (OR = 0.41; 95% CI: 0.17, 1.00). In contrast, liquor drinkers consuming one or more drinks per day had more than 2-fold higher risk of PD than non-alcohol drinkers. No association was observed between exclusive drinkers of wine and risk of PD.

**Table 3:** Odds Ratios of Parkinson’s Disease According to Exclusive Drinkers of a Specific Type of Alcoholic Beverage, NIH-AARP Diet and Health Study, 1995–2006.

		Model 1^a^	Model 2^b^
Drinks/day	PD/No PD	OR (95% CI)	OR (95% CI)
Beer drinkers only			
None	262/66,700	1.00	1.00
<1	39/11,163	0.90 (0.65, 1.27)	0.83 (0.59, 1.17)
≥1	5/3,267	0.42 (0.17, 1.01)	0.41 (0.17, 1.00)
P for trend		0.05	0.05
Wine drinkers only			
None	262/66,700	1.00	1.00
<1	86/23,573	0.93 (0.73, 1.19)	1.08 (0.84, 1.38)
≥1	7/1,982	0.87 (0.41, 1.84)	1.10 (0.52, 2.35)
P for trend		0.65	0.75
Liquor drinkers only			
None	262/66,700	1.00	1.00
<1	24/7,469	0.85 (0.56, 1.29)	1.01 (0.66, 1.54)
≥1	10/1,291	1.85 (0.98, 3.49)	2.18 (1.14, 4.17)
P for trend		0.06	0.02

CI = confidence interval; OR = odds ratio; PD = Parkinson’s disease.

a Adjusted for age.

b Additional adjustment for sex, race, education, marital status, smoking, caffeine intake, physical activity, and self-evaluated health status.

**Figure 1:**
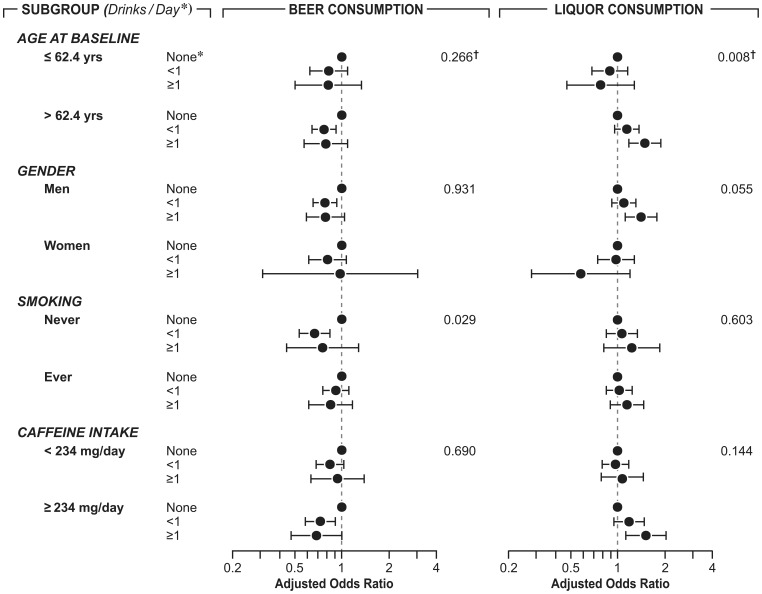
Odds ratios and 95% confidence intervals of Parkinson’s disease according to beer and liquor consumption within subgroups, NIH-AARP Diet and Health Study, 1995–2006. † indicates p for interaction.

Finally, we conducted an exploratory analysis to examine whether the association between beer or liquor consumption and risk of PD was modified by age, sex, smoking status, and caffeine intake (**Figure 1**). Generally, the analyses revealed no clear effect modifications. Although a statistically significant interaction was found between beer consumption and smoking, no clear trends were observed. For liquor, a higher PD risk was observed with increasing liquor consumption for those aged 62.4 year and older than younger individuals (P for trend = 0.003). A marginal significant interaction (P = 0.055) was observed between gender and liquor consumption with a positive association in men (P for trend = 0.005) but not in women (P for trend = 0.1). Finally, visual inspections of the figure suggest that when stratified by caffeine intake, the association of beer or liquor with PD only exists among individuals with higher caffeine intake. However, the P for interaction was not statistically significant.

## Discussion

In this large prospective cohort study of older adults, we did not find evidence for an association between total alcohol consumption and risk of PD. However, a differential effect was observed for different types of alcoholic beverages. While low to moderate beer consumption may be associated with lower PD risk, greater liquor consumption was associated with higher PD risk. Wine consumption did not appear to be associated with risk of PD.

The role of alcohol drinking in PD etiology has not been extensively evaluated. Most of the previous epidemiologic studies were case-control studies and did not differentiate the specific types of alcoholic beverage, and most did not find an association between alcohol consumption and PD risk. The few existing prospective studies also showed inconsistent results. Detailed study design and results of previous publications are summarized in **Table S1**. Our finding that low to moderate beer consumption and lower PD risk is consistent with that from the Health Professionals Follow-up Study and the Nurses’ Health Study [17], while result on liquor consumption was different. Results from the recent Cancer Prevention Study II Nutrition Cohort, however, found no association with any types of alcohol beverages [18]. In the Swedish twins study, alcohol intake in general was not associated with PD risk; however, stratified analyses among never smokers showed a lower PD risk for ever drinkers as compared with abstainers [19]. The discrepant results with alcoholic beverages observed across studies may be due to differences in study design, dietary assessment, and adjustment for potential confounders (e.g. smoking and coffee consumption). For example, the majority of the studies are case-control studies with prevalent cases which might be more prone to recall biases and reverse causation.

Biological mechanisms that link alcohol and PD are unclear and speculative. Our finding of differential associations with specific types of alcoholic beverages suggests mechanisms involving factors other than or in addition to ethanol itself. Beer, but not wine or liquor, contains a large amount of purine, which may work synergistically with ethanol to augment plasma urate [6]. Urate is a potent free-radical scavenger [20], and has been linked to lower PD risk [7]–[10] and slower clinical progression among PD patients [11], [12]. Relative to wine or liquor, beer also contains high levels of niacin [21], which has been reported to reduce the risk of PD [22]. Our finding of higher PD risk among heavy liquor drinkers is more difficult to explain. We speculate this may be related to the relatively high proportion of pure ethanol found in liquor compared to both wine and beer [23]. Pure ethanol has been shown to induce oxidative stress by acting as a pro-oxidant and may be proinflammatory [24]. Moreover, liquor and other distilled beverages contain no vitamins or antioxidants [21]. Polyphenolic components (e.g., resveratrol) found in red wine has been shown to attenuate neurotoxin 6-hydroxydopamine (6-OHDA)-induced toxicity in animal models of PD [25], [26] via its antioxidant and anti-inflammatory potentials. However, existing epidemiologic studies including ours have largely failed to find a neuroprotective effect of wine on risk of PD.

Major strengths of the current study include the prospective data collection, large sample size, examination of total and specific types of alcoholic beverages, and stratified analyses by PD risk factors. Our study also has several limitations. We only assessed the frequency and portion size of alcohol consumption in the past 12 months prior to baseline, therefore we were unable to examine lifetime alcohol consumption or drinking patterns in relation to PD risk. Further, exposure misclassification was likely; if this misclassification was random, it would attenuate the true relationship. In addition, due to the observational nature of this analysis, we could not exclude the possibility of residual or unmeasured confounding. Nevertheless, we tried to control for a variety of socio-economic variables and conducted stratified analyses by known PD risk factors. In terms of PD assessment, we had to rely on self-reported diagnoses to identify potential PD patients in this large US cohort, and this inevitably introduced reporting and diagnostic errors. However, our validation study validated 88% of the self-reported diagnoses among whom medical information was available. We further removed from the analysis persons with identified erroneous reports or misdiagnoses. Finally, our analysis was conducted only among participants in the follow-up survey and therefore we could not exclude the possibility of selection bias due to loss to follow-up.

In conclusion, our findings suggest that total alcohol consumption is not related to the risk of PD, but specific types of alcoholic beverages may have different effects on PD risk. Our observation of lower PD risk among low to moderate beer drinkers but higher PD risk among heavy liquor drinkers warrant further investigations.

## Supporting Information

Table S1 **Characteristics of Studies on the Association between Alcohol Consumption and Parkinson’s Disease.**
(DOC)
