# Supplementary material for: Alcohol Consumption, Types of Alcohol, and Parkinson’s Disease
Source: PLoS One. 2013 Jun 19;8(6):e66452. doi: 10.1371/journal.pone.0066452 (PMC3686735; doi:10.1371/journal.pone.0066452)
Supplement: Table S1 — Characteristics of Studies on the Association between Alcohol Consumption and Parkinson’s Disease. (DOC) [file pone.0066452.s001.doc]

**Table S1.** Characteristics of Studies on the Association between Alcohol Consumption and Parkinson’s Disease.

| First author, year | Study  Design | | Population / location | | PD identification | Controls | | Exposure assessment | | Effect estimate (95% CI) | | | Comments |
| --- | --- | --- | --- | --- | --- | --- | --- | --- | --- | --- | --- | --- | --- |
|  |  | |  | |  |  | |  | | Primary Overall Results | Types of Alcohol | |  |
| ***Prospective studies*** | | | | | | | | | | | | | |
| Paganini-Hill,  2001 [1] | Nested case-control | | 395 PD cases  2,320 matched-controls  US | Incident PD  Hospital discharge diagnoses, death certificates, and self-report of physician diagnosed PD | | Six matched controls per case | | Self-administered questionnaire; daily alcohol consumption (wine, beer, hard liquor) | Highest alcohol consumption  (2+ drinks/day) vs. Nondrinkers:  OR=0.77 (0.58-1.03) | | Wine:  OR=0.80 (0.51-1.24)  Beer:  OR=0.32 (0.10-1.06)  Hard Liquor:  OR=0.75 (0.55-1.01) | | Adjusted for smoking, blood pressure medication, number of children, coffee, dietary vitC and total vitA.  No gender-specific analyses |
| Hernán,  2003 [2] | Cohort | | 415 incident PD cases from two prospective cohorts  US | Incident PD  Self-report followed by physician confirmation | |  | | SFFQ;  Frequency of consumption of beer, wine, and liquor in the past year | Highest alcohol consumption  (≥30gm/day) vs. Nondrinkers:  OR=0.7 (0.5-1.2) | | Beer:  ≥1/wk vs. <1/mo:  OR=0.7 (0.5-0.9)  Wine:  ≥5/wk vs. <1/mo.  OR=1.1 (0.8-1.7)  Liquor:  ≥5/wk vs. <1/mo.  OR=1.1 (0.8-1.6) | | Adjusted for age, pack-yr smoking, caffeine intake  Men: ≥30gm/day vs. nondrinkers:  OR=0.6 (0.4-1.1)  Women: ≥30gm/day vs. nondrinkers:  OR=1.0 (0.4-2.2) |
| Hernán,  2004 [3] | Nested case-control | | 1,019 PD cases  10,123 age, sex, and start date-matched controls  United Kingdom | Incident PD  Computerized medical records | | Randomly selected up to 10 controls per case | | Clinically-defined alcoholics: computerized diagnosis of alcoholism or alcohol-related disease | Alcoholics vs. Non-alcoholics  OR=1.09 (0.67-1.78)  Highest alcohol consumption  (≥50units/wk) vs. Nondrinkers:  OR=1.46 (0.69-3.01) | | Type(s) of alcohol not specified | | Adjusted for smoking  Men:  OR=0.83 (0.46-1.51)  Women:  OR=2.74 (1.10-6.82) |
| Wirdefeldt,  2005 [4] | Nested case-control | | 476 PD cases 2,380 external controls matched by sex, birth yr, and questionnaire source of exposure data;  and 415 same-sex twin pairs  Sweden | Incident PD  Identified from the Swedish Inpatient Discharge Register (IDR) and the Cause of Death Register (CDR) | | Two types of controls (external control and co-twin control) | | Questionnaire | Alcohol consumption was categorized into never and ever drinkers, and also computed total alcohol intake/day (in grams) for beer, wine or spirits  Ever vs. Never (crude OR)  OR=0.74 (0.59-0.93)  E*xternal control* comparison  Highest alcohol consumption)  (>30gm/day) vs. Nondrinkers:  OR=0.66 (0.34-1.29)  *Co-twin* comparison  Highest alcohol consumption  (>30gm/day) vs. Nondrinkers:  OR=0.72 (0.19-2.65) | | Type(s) of alcohol not specified | | Adjusted for smoking, coffee, and education  Men:  Highest alcohol consumption (E*xternal control* comparison)  (>30gm/day vs. nondrinkers):  OR=0.68 (0.29-1.59)  Highest alcohol consumption (*Co-twin* comparison)  (>30gm/day vs. nondrinkers):  OR=0.44 (0.06-3.35)  Women:  Highest alcohol consumption (E*xternal control* comparison)  (>30gm/day vs. nondrinkers):  OR=0.65 (0.21-2.04)  Highest alcohol consumption (*Co-twin* comparison)  (>30gm/day vs. nondrinkers):  OR=0.60 (0.07-5.21) |
| Palacios,  2012 [5] | Cohort | | 605 incident PD cases (389 male and 216 female)  US | Incident PD  Self-report followed by physician or medical record confirmation | |  | | Modified Block FFQ;  Frequency of consumption of beer, wine or wine cooler, and liquor in the past year | Highest alcohol consumption for Men:  (≥30gm/day) vs. Nondrinkers:  OR=1.29 (0.90-1.86)  for Women:  (≥15gm/day) vs. Nondrinkers:  OR=0.77 (0.41-1.45) | | 10gm/day increments in consumption of (men and women combined)  Beer:  OR=1.01 (0.99-1.03)  Wine:  OR=1.02 (0.98-1.05)  Liquor:  OR=0.99 (0.96-1.02) | | Adjusted for age, smoking, and coffee intake. |
| ***Case-control studies*** | | | | | | | | | | | | | |
| Ho,  1989 [6] | Case-control | 35 cases  105 age- and sex matched controls  Hong Kong | | | Prevalent PD  Hospital PD patients and PD patients identified from survey of elderly home residents | | Elderly homes and hospital controls | In-person interview with structured questionnaire | Ever vs. Never drinkers  OR=0.7(0.2-2.0) | | | Type(s) of alcohol not specified | Types of adjustments not specified, likely crude OR  No gender-specific results |
| Jiménez-Jiménez,  1992 [7] | Case-control | 128 PD cases  256 age-and sex-matched controls  Spain | | | Prevalent PD  Outpatients from movement disorder clinic | | Patients in ER of same hospital with minor non-neurological ailments | In-person interview | Light drinkers vs. Nondrinkers: (<50 g/d): p<0.0001; no OR reported | | | Type(s) of alcohol not specified | Correlation study  Men: drinking ≥50g/day was less frequent in PD than controls (p<0.001)  Women: drinking behavior did not differ significantly between PD than controls |
| Wang,  1993 [8] | Case-control | 93 PD cases  186 age-, sex-, and hospital matched controls  China | | | Prevalent PD  Randomly selected neurology outpatients | | Randomly selected from the same hospital with other diagnoses unrelated to PD | Questionnaire, interview | Drinking (<50g/d): OR=0.28 (012-0.65) | | | Hard liquor: OR=0.60 (0.36-0.99) | Types of adjustments not specified, likely crude OR  No gender-specific analyses |
| Hellenbrand,  1996 [9] | Case-control | 342 PD cases and 342 controls  Germany | | | Prevalent PD  Recruited from multiple neurologic clinics | | Randomly selected from the same neighborhood or region (two random community controls per case) | Structured interview and self-administered FFQ | No overall alcohol consumption reported | | | Beer  (quartile 4 vs. quartile 1): OR=0.26 (0.14-0.49)  Spirits  (quartile 4 vs. quartile 1): OR=0.56 (0.36-0.86)  Wine  Quartile 2: OR=1.20 (0.74-1.94)  Quartile 3: OR=1.14 (0.70-1.85)  Quartile 4: OR=1.20 (0.71-2.04)  Ethanol  Quartile 2: OR=1.14 (0.71-1.84)  Quartile 3: OR=0.53 (0.30-0.93)  Quartile 4: OR=0.58 (0.32-1.06) | Adjusted for energy intake, smoking in pack years, and education |
| Liou,  1997 [10] | Case-control | 120 PD cases  240 age- and sex-matched hospital controls  Taiwan | | | Prevalent PD  Recruited from movement disorder clinic | | Recruited from the neurologic or medical outpatient clinics at the same hospital | In-person interview with structured open-ended questionnaire | Ever vs. Never  OR=0.59 (0.26-1.33) | | | Type(s) of alcohol not specified | Types of adjustments not specified, likely crude OR  No gender-specific analyses |
| Smargiassi,  1998 [11] | Case-control | 86 PD cases  86 controls  Italy | | | Prevalent PD  Consecutive cases from neurology institute | | Outpatients of the same hospital | Interview with structured questionnaire;  Alcohol drinkers were those consuming >50 g/day on average during lifetime | Ever vs. Never  OR=1.90 (0.27-4.79) | | | Type(s) of alcohol not specified | Types of adjustments not specified, likely crude OR  No gender-specific analyses |
| Gorell,  1999 [12] | Case-control | 144 PD cases and 464 age-, sex-, race- frequency matched controls  US | | | Prevalent PD  Drawn from health system database | | Drawn from health system database | Telephone interview | Alcohol consumption were classified as nondrinkers (0 drink-yrs), mild to moderate (>0 to 10 drink-yrs), or heavy (>10 drink-yrs)  “Drink-yr” was defined as the intake of 1drink of alcohol/day for 1 yr.  Never drank: 1 (reference)  Mild-moderate drinker: OR=0.74 (0.41-1.35)  Heavy drinker: OR=0.94 (0.51-1.75) | | | Type(s) of alcohol not specified | Types of adjustments not specified, likely crude OR  No gender-specific analyses |
| Fall,  1999 [13] | Case-control | 113 PD cases and 263 controls  Sweden | | | Prevalent PD  Drawn from prescription records and general practitioner reports | | Randomly drawn from the population register | Structured questionnaire | No overall alcohol consumption reported | | | Medium strong beer  (1-3 bottles/day vs. ≤1, never)  OR=0.47 (0.26-0.83)  Stronger beer  (1 bottles/wk vs. <1, never)  OR=0.38 (0.15-0.90)  Wine  (2-6 bottles/day vs. never)  OR=0.25 (0.11-0.56)  Liquor  ½-1 bottle/mo:  OR=0.51 (0.27-0.93)  ½ bottle/wk;  OR=0.19 (0.06-0.52)  ≥1 bottle/wk: OR=0.24 (0.06-0.80) | Types of adjustments not specified, likely crude OR  No gender-specific analyses |
| Benedetti,  2000 [14] | Case-control | 196 PD cases  196 age- and sex-matched population control  US | | | Incident PD  Identified from medical records linkage system | | Selected randomly from the same county population | Extracted from medical records | Ever vs. Never:  OR=1.04 (0.61-1.76)  Regular vs. Otherwise (occasional, rare, never):  OR=1.00 (0.64-1.57)  Heavy vs. Otherwise (moderate, light, never):  OR=0.65 (0.30-1.38)  Alcoholism (Ever vs. never)  OR= 0.41 (0.19-0.89) | | | Type(s) of alcohol not specified | Adjusted for education, smoking, and coffee drinking  Controls subjects were diagnosed with alcoholism significantly more frequently than cases  No gender-specific analyses |
| Behari,  2001 [15] | Case-control | 377 PD cases and 377 age-matched controls  India | | | Incident and prevalent PD  Consecutive PD patients attending movement disorder clinic | | Outpatients with other neurological diseases attending same medical institute | In-person interview with standard structured questionnaire;  Alcohol was considered positive if a subject consumed 30ml of alcohol /day for at least 1 yr | Highest consumption (>20 yrs) vs. None: OR=1.48 (0.82-2.65) | | | Type(s) of alcohol not specified | Types of adjustments not specified, likely crude OR  No gender-specific analyses |
| Checkoway,  2002 [16] | Case-control | 210 PD cases and 347 age-and sex-matched controls  US | | | Incident PD  Diagnosis logs, pharmacy database, and chart reviews | | Health cooperative enrollees without PD or other progressive neurologic | In-person questionnaire | Highest consumption (≥10 drinks/wk) vs. None:  OR=0.8 (0.4-1.4) | | | Type(s) of alcohol not specified | Adjusted for age, ethnicity, gender, and education  No gender-specific analyses |
| Ragonese,  2003 [17] | Case-control | 150 PD cases and 150 age-, sex-and location- matched controls  Italy | | | Prevalent PD  Consecutive outpatients at neurological clinics | | Random selected population records of the municipality | Structured questionnaire | Ever vs. Never:  OR=0.61 (0.43-0.89)  Yrs of alcohol drinking  None:1.0 (reference)  <30: OR=0.25 (0.08-0.70)  >30: OR=0.88 (0.52-1.49)  Drink-yrs  None: 1.0 (reference)  1-6: OR=0.37 (0.03-2.46)  >6-36.5: OR=0.57 (0.29-1.12)  >36.5: OR=0.84 (0.44-1.57) | | | No alcohol-specific analyses | Adjusted for education, coffee drinking, and cigarette smoking  Men: OR=0.55 (0.23-1.35)  Women: OR=0.56 (0.28-1.13) |
| Tan,  2003 [18] | Case-control | 200 PD case and control pairs matched for age, gender, and race  China | | | Prevalent PD  Randomly selected from referral hospital movement disorder database | | Participants in community health screening programme | Screening questionnaire, interview | Cup-years (average number of cups/day x number of years of drinking); a cup of beer was used as reference  OR reported to be significantly lower than 1; exact OR (CIs) not reported. | | | Type(s) of alcohol not specified | Adjusted for smoking, tea, coffee, head injury, stroke, hypertension, presence of heart conditions, toxin exposure, and farm dwelling  No gender-specific analyses |
| Evans,  2006 [19] | Case-control | 106 PD cases  106 age- and sex- matched healthy controls  UK | | | Prevalent PD  Consecutive outpatients of Caucasian decent | | Recruited form the same outpatient department as cases | SFFQ | Highest consumption (≥14 units/day)  OR=0.58 (0.34-0.98) | | | Type(s) of alcohol not specified | Adjusted for sensation seeking score (SSS)  No gender-specific analyses |
| Dicks,  2007 [20] | Case-control | 767 PD cases and 1,989 age- and sex-matched controls  Italy | | | Prevalent PD  PD cases identified from five centers and includes:  consecutive hospital outpatients, patients receiving L-dopa treatment, medical records reviews of outpatient clinics, and clinic lists | | Recruited from anticoagulant clinics, hospital inpatients, community, and a mixture of community controls and hospital outpatients | Interviewer-administered questionnaire | No overall alcohol consumption reported | | | Ever consumed beer, wine or spirits regularly vs. never  OR=1.01 (0.83-1.23) | Adjusted for age, sex, country, ever used tobacco-containing product, ever knocked unconscious and first-degree family history of PD |
| Dhillon,  2008 [21] | Case-control | 102 PD cases and 84 controls  US | | | Prevalent PD  PD patients from neurology institute | | Selected from the same medical practice | Standardized questionnaire;  Alcohol intake in the last 12 months | Highest consumption (>once/wk) vs. None:  OR=0.5 (0.2-1.1) | | | Type(s) of alcohol not specified | Types of adjustments not specified, likely crude OR  No gender-specific analyses |
| Brighina,  2009 [22] | Case-control | 893 PD case- controls pairs  US | | | Prevalent PD  Hospital based PD patients | | Two types of controls: unaffected siblings (n=514) and unrelated controls (n=379) | Structured telephone interview and screened for alcohol use disorders using the CAGE questionnaire | Ever vs. Never  OR= 0.88 (0.69-1.22) | | | Beer (ever vs. never):  OR= 0.96 (0.75-1.23)  Wine (ever vs. never):  OR= 1.19 (0.95-1.50)  Liquor (ever vs. never):  OR= 0.83 (0.67-1.02) | Adjusted for age, sex, education, smoking and coffee |
| Fukushima,  2010 [23] | Case-control | 214 PD cases  327 controls  Japan | | | Prevalent PD  Hospital based PD patients | | Inpatients and outpatients w/o neurodegenerative disease | Self-administered questionnaires;  daily & weekly peak consumption | Highest consumption (≥6 days/wk) vs. Nondrinkers:  OR=0.96 (0.50-1.81)  Ethanol g/day  Nondrinker: 1.0 (reference)  0.1-65.9: OR=1.07 (0.64-1.80)  ≥66: OR=1.46 (0.79-2.71)  Ethanol g/wk  Nondrinker: 1.0 (reference)  0.1-219.3: OR=0.98 (0.58-1.65)  ≥219.4: OR=1.79 (0.95-3.39) | | | Highest consumption (≥66 g ethanol/day) vs. non-drinkers  Beer:  OR=2.13 (0.80-5.82)  Japanese sake: OR=3.39 (1.10-11.0)  Shochu:  OR=1.29 (0.59-2.78)  Wine:  OR=6.11 (0.67-1.34)  Whisky:  OR=2.25 (0.67-7.83) | Adjusted for sex, age, region of residence, smoking, education, BMI, alcohol flushing status, medication history, caffeine, and multiple dietary factors  No gender-specific analyses |
| Nicoletti,  2010 [24] | Case-control | 492 PD cases and 459 controls  Italy | | | Prevalent PD  PD patients from movement disorder centers | | Two groups of controls: 1) spouse of enrolled patient and 2)subjects who accompanied non-parkinsonian patients for hospital check-ups | In-person interview using standardized structured questionnaire | No overall alcohol consumption reported (restricted analysis to wine consumption) | | | Wine consumption  (≥3 glasses/day vs. none)  OR= 0.45 (0.28-0.74)  Years of wine drinking  (≥46yrs vs. none)  OR=0.45 (0.29-0.68) | Adjusted for age, sex, family history, place of residence, coffee consumption, and smoking  No gender-specific analyses |

Abbreviations: FFQ = food-frequency questionnaire; SFFQ = semiquantitative food frequency questionnaire; OR= odds ratio; PD = Parkinson’s disease.

**SUPPLEMENTARY REFERENCES**

1. Paganini-Hill A (2001) Risk factors for parkinson's disease: the leisure world cohort study. Neuroepidemiology 20: 118-124.

2. Hernán MA, Chen H, Schwarzschild MA, Ascherio A (2003) Alcohol consumption and the incidence of Parkinson's disease. Ann Neurol 54: 170-175.

3. Hernán MA, Logroscino G, Rodriguez LA (2004) A prospective study of alcoholism and the risk of Parkinson's disease. J Neurol 251 Suppl 7: vII14-17.

4. Wirdefeldt K, Gatz M, Pawitan Y, Pedersen NL (2005) Risk and protective factors for Parkinson's disease: a study in Swedish twins. Ann Neurol 57: 27-33.

5. Palacios N, Gao X, O'Reilly E, Schwarzschild M, McCullough ML, et al. (2012) Alcohol and risk of Parkinson's disease in a large, prospective cohort of men and women. Mov Disord 27: 980-987.

6. Ho SC, Woo J, Lee CM (1989) Epidemiologic study of Parkinson's disease in Hong Kong. Neurology 39: 1314-1318.

7. Jimenez-Jimenez FJ, Mateo D, Gimenez-Roldan S (1992) Premorbid smoking, alcohol consumption, and coffee drinking habits in Parkinson's disease: a case-control study. Mov Disord 7: 339-344.

8. Wang WZ, Fang XH, Cheng XM, Jiang DH, Lin ZJ (1993) A case-control study on the environmental risk factors of Parkinson's disease in Tianjin, China. Neuroepidemiology 12: 209-218.

9. Hellenbrand W, Seidler A, Boeing H, Robra BP, Vieregge P, et al. (1996) Diet and Parkinson's disease. I: A possible role for the past intake of specific foods and food groups. Results from a self-administered food-frequency questionnaire in a case-control study. Neurology 47: 636-643.

10. Liou HH, Tsai MC, Chen CJ, Jeng JS, Chang YC, et al. (1997) Environmental risk factors and Parkinson's disease: a case-control study in Taiwan. Neurology 48: 1583-1588.

11. Smargiassi A, Mutti A, De Rosa A, De Palma G, Negrotti A, et al. (1998) A case-control study of occupational and environmental risk factors for Parkinson's disease in the Emilia-Romagna region of Italy. Neurotoxicology 19: 709-712.

12. Gorell JM, Rybicki BA, Johnson CC, Peterson EL (1999) Smoking and Parkinson's disease: a dose-response relationship. Neurology 52: 115-119.

13. Fall PA, Fredrikson M, Axelson O, Granerus AK (1999) Nutritional and occupational factors influencing the risk of Parkinson's disease: a case-control study in southeastern Sweden. Mov Disord 14: 28-37.

14. Benedetti MD, Bower JH, Maraganore DM, McDonnell SK, Peterson BJ, et al. (2000) Smoking, alcohol, and coffee consumption preceding Parkinson's disease: a case-control study. Neurology 55: 1350-1358.

15. Behari M, Srivastava AK, Das RR, Pandey RM (2001) Risk factors of Parkinson's disease in Indian patients. J Neurol Sci 190: 49-55.

16. Checkoway H, Powers K, Smith-Weller T, Franklin GM, Longstreth WT, Jr., et al. (2002) Parkinson's disease risks associated with cigarette smoking, alcohol consumption, and caffeine intake. Am J Epidemiol 155: 732-738.

17. Ragonese P, Salemi G, Morgante L, Aridon P, Epifanio A, et al. (2003) A case-control study on cigarette, alcohol, and coffee consumption preceding Parkinson's disease. Neuroepidemiology 22: 297-304.

18. Tan EK, Tan C, Fook-Chong SM, Lum SY, Chai A, et al. (2003) Dose-dependent protective effect of coffee, tea, and smoking in Parkinson's disease: a study in ethnic Chinese. J Neurol Sci 216: 163-167.

19. Evans AH, Lawrence AD, Potts J, MacGregor L, Katzenschlager R, et al. (2006) Relationship between impulsive sensation seeking traits, smoking, alcohol and caffeine intake, and Parkinson's disease. J Neurol Neurosurg Psychiatry 77: 317-321.

20. Dick FD, De Palma G, Ahmadi A, Scott NW, Prescott GJ, et al. (2007) Environmental risk factors for Parkinson's disease and parkinsonism: the Geoparkinson study. Occupational and Environmental Medicine 64: 666-672.

21. Dhillon AS, Tarbutton GL, Levin JL, Plotkin GM, Lowry LK, et al. (2008) Pesticide/environmental exposures and Parkinson's disease in East Texas. J Agromedicine 13: 37-48.

22. Brighina L, Schneider NK, Lesnick TG, de Andrade M, Cunningham JM, et al. (2009) Alpha-synuclein, alcohol use disorders, and Parkinson disease: a case-control study. Parkinsonism Relat Disord 15: 430-434.

23. Fukushima W, Miyake Y, Tanaka K, Sasaki S, Kiyohara C, et al. (2010) Alcohol drinking and risk of Parkinson's disease: a case-control study in Japan. BMC Neurol 10: 111.

24. Nicoletti A, Pugliese P, Nicoletti G, Arabia G, Annesi G, et al. (2010) Voluptuary habits and clinical subtypes of Parkinson's disease: the FRAGAMP case-control study. Mov Disord 25: 2387-2394.
